# Hydrocyanate of Iron in the Treatment of Neuralgia

**Published:** 1882-08-20

**Authors:** Charles K. Gardner

**Affiliations:** Laurinburg, N. C.


### HYDROCYANATE OF IRON IN THE TREATMENT OF NEURALGIA.

BY CHAS. K. GARDNER, M. D., LAURINBURG, N. C.

For several months prior to my experience of the therapeutic effect of the above agent in the treatment of this malady, my attention was directed to an interesting article published in the American Medical Bi-Weekly, Vol. XIII, No. 9, lauding its efficacy, and without a previous knowledge of the medicine, it being non-officinal, I determined its trial in my next case of neuralgia.

Mrs. R., æt. 47, generally anæmic, with every existing symptom of phthisis, had long been an extreme sufferer of periodical attacks of neuralgia, often of a week's duration, always supervening upon the slightest meteorologic change. These exacerbations continued throughout the period of inclemency, with but trivial amelioration, despite the myriads of domestic remedies considered so potent for relief among the non-professional. As ascertained, no physician had been consulted for several years, all attempts at cure having proved futile; and had there not been an aggravated condition of the pulmonary symptoms, the result of undue exposure, cough, slight pain on respiration, etc., my presence at the bedside, in the patient's estimation, would have been unnecessary, for she had long since abandoned the assistance art endeavored to afford in the treatment of her obstinate case; and on making inquiry upon which to base my diagnosis, I was told, that although suffering with neuralgic pains of the head and face, my presence was desired only to afford relief to the pulmonary symptoms, the former being of such frequent recurrence, and from which she had so long suffered, that all treatment had proven futile, being almost content to endure the pangs of torture awaiting a spontaneous recovery.

For the lung symptoms I only prescribed an anodyne cough mixture, using upon the thorax oleum tiglii to pustulation. My attention was then transferred to the neuralgic lesion. The pain radiated from the anterior aspect of the right ear across the cheek, involving the globe of the eye and upper lid; the auriculo-temporal branch of the inferior maxillary and the ophthalmic branch divisions of the fifth pair of nerves being evidently involved. Assuring my patient of the much hope I entertained of her recovery, and having preceded my treatment by mild catharsis, I ordered the following:

℞ Ferri hydrocyanatis, } aa ℥ ss
Quiniæ sulph }
Strychniæ . . . . . . . . gr. ss,
Ext. belladonnæ . . . . . . . . gr. v,
Ext. gentianæ . . . . . . . . q. s.
M. Ft. pil. No. xxx.
Sig. One before each meal.

The next evening I visited the patient, four pills having been taken. Found a decided improvement; pain ameliorated; patient unusually quiescent, with a tendency to sleep. This treatment was pursued until there was a final disappearance of the neuralgia, which, despite its periodic nature during inclement weather, did not return, eighty pills, or the part of three prescriptions, having effected a cure. During this course of treatment, no untoward symptoms resulted from the sedative or depressing influence of the hydrocyanate, the appetite being increased, and digestion more vigorous under its tonic properties.

In conclusion, this valuable medicine should receive its merited encomium, by being consigned to the physician's armamentarium as potent for the relief of this painful disease.—*Southern Clinic.*

